# Does Elective Sternal Plating Combined with Steel Wire Reduce Sternal Complication Rates in Patients with Obesity?

**DOI:** 10.21470/1678-9741-2022-0089

**Published:** 2023

**Authors:** Ersin Çelik, Ahmet Rıfkı Çora

**Affiliations:** 1 Department of Cardiovascular Surgery, Isparta City Hospital, Isparta, Turkey

**Keywords:** Sternotomy, Sternal Dehiscence, Sternal Closure, Titanium, Sternal Infection

## Abstract

**Introduction:**

In this study, sternal complication rates of sternal closures with steel wire or steel wire combined with titanium plate in patients with obesity that underwent cardiac surgery were investigated.

**Methods:**

The data of 316 patients that underwent cardiac surgery between May 2018 and October 2021 were analyzed retrospectively; 124 patients withbody mass index (BMI) ≥ 30 kg/m^2^ were divided into group I, patients whose sternotomy was performed with steel wires, and group II, patients whose sternotomy was performed with steel wire combined with titanium plates.

**Results:**

A total of 124 patients with BMI ≥ 30 kg/m^2^ were divided into group I (n=88 [70.9%]) and group II (n=36 [29.1%]). The rate of male patients was found to be significantly higher in group I, whereas the rate of female patients was significantly higher in group II (*P*<0.001). BMI values were found to be low in group I and high in group II (*P*<0.001). The distribution of complications was different in the BMI ≥ 35.00-39.99 kg/m^2^ and ≥ 40 kg/m^2^ groups (*P*=0.003). Development of complications was found to be higher in patients with BMI ≥ 40 kg/m^2^. Sternal dehiscence was observed in two patients in group I, while no dehiscence was observed in group II.

**Conclusion:**

The lower incidence of complications and the absence of non-infectious sternal complications and sternal dehiscence in patients with BMI ≥ 35 kg/m^2^ that underwent steel wire combined titanium plate sternal closure strengthened the idea that plate-supported sternal closure can prevent sternal complications in high-risk patients.

## INTRODUCTION

Nowadays, median sternotomy is still the most commonly used approach to reach the heart in cardiac surgeries^[1,2]^. Despite the advances in cardiac surgery, median sternotomy has remained as a potential cause of significant morbidity and mortality, in addition to being costly. Although major complications such as sternal dehiscence, mediastinitis, superficial and deep sternal wound infection, and osteomyelitis following sternotomy are seen rarely (0.5-2.5%), the mortality rate varies between 10% and 40%^[1,3,4]^. The risk factors that contribute to these complications the mostinclude body mass index (BMI) ≥ 30kg/m^2^, male gender, advanced age, tobacco use, chronic obstructive pulmonary disease (COPD), prolonged mechanical ventilation, steroid dependence, New York Heart Association (or NYHA) class IV status, surgical priority, low ejection fraction (EF), diabetes mellitus (DM), osteoporosis, renal failure, peripheral vascular disease, bilateral internal mammary artery harvesting, surgical reexploration, long cardiopulmonary bypass (CPB) time, asymmetric sternotomy, sepsis, and respiratory failure^[4,5,6]^.

Good sternal fixation is essential to avoid complications after sternotomy. In general, high strength of the sternum closure technique and the adequate stifiness it provides are suficient to prevent the development of dehiscence^[7,8]^. Steel wire closure is the most commonly used technique for sternal closure after median sternotomy^[9]^. However, sternal closure has been applied by using many other different materials and techniques until today. Yet, there is no consensus on the best technique^[7,8,9,10]^. Recently, enhanced sternal stabilization (Class of Recommendation: IIb, Level of Evidence: C) has been recommended for high-risk patients by The Society of Thoracic Surgeons Clinical Practice Guidelines^[11]^. In this study, we aimed to investigate sternal complication rates in sternal closures with steel wire or steel wire combined with titanium plate in patients with obesity that underwent cardiac surgery.

## METHODS

### Patient Data

After approval was obtained from Suleyman Demirel University Ethics Committee (number 23.12.2021/367), the data of patients that underwent cardiac surgery between May 2018 and October 2021 were analyzed retrospectively. Demographic, clinical, operative, and outcome data of all the median sternotomy patients with BMI ≥ 30kg/m^2^ were also collected retrospectively. Written informed consent was obtained from each patient. All of the patients had previously been operatedata single institution (Isparta City Hospital, Isparta, Turkey) by three surgeons that were experts in adult cardiac surgery. Patients that underwent partial or redo sternotomy were excluded.

### Clinical Outcomes and Definitions

Patients were divided into two groups as group I, patients whose sternotomy was performed with steel wires, and group II, patients whose sternotomy was performed with steel wire combined with titanium plates. Detailed medical history review, physical examination and routine blood tests, echocardiogram, electrocardiogram, carotid doppler ultrasonography, chest X-ray and respiratory function tests, and BMI calculation were performed for all patients scheduled to undergo open-heart surgery. The patients were evaluated in terms of demographic dataandrisk factors for sternal dehiscence, such as BMI, DM, COPD, and smoking. The patients that were tobacco consumers at the time of coronary angiography were considered as smokers. The diagnosis of DM was confirmed with internal medicine consultation requested both for the patients that had a previous diagnosis of DM and for those who did not have a diagnosis of DM but had fasting blood glucose > 126 mg/dl. All patients that had COPD were evaluated by a chest physician with a pulmonary function test or arterial blood gas test, and those who could not perform pulmonary function tests were evaluated with the results of a physical examination. For patients that were diagnosed with COPD, respiratory physiotherapy was applied under the guidance of a physiotherapist in the cases where forced expiratory volume in the first second/forced vital capacity was found to be < 70% in pulmonary function tests or oxygen saturation on room air was found to be < 94% in the fingertips of patients that could not perform a pulmonary function test. A three-ball spirometer was used in all the patients in the preoperative period. CPB times were evaluated based on perioperative data. Patients were evaluated in terms of the amount of blood and the blood products used in the postoperative period, the length of intensive care unit (ICU) and hospital stays, non-infectious sternal wound complications, deep sternal infection, and development of sternal dehiscence. The primary endpoint of the study was the development of sternal complications. Sternal dehiscence was identified as an existence of a palpable sternal click on physical examination or radiographic evidence of nonunion or wire fracture. Deep sternal wound infection was identified as dehiscence of the sternal wound or pectoral myofascial layer, and any wound infection that required intravenous antibiotics over 48 hours. Non-infectious sternal wound complications were defined either as a complaint of discomfort due to hardware, or a persistent reactive tissue inflammation that requires an operative intervention. Outcomes of sternal wound complications of the patients that underwent traditional wire cerclage closure and sternal plate reinforcement were compared.

### Operative Technique

All patients were bathed with a solution that contained 2% chlorhexidine gluconate one night before the operation. Prophylactically, 60 minutes before the operation, 1 g of cefazolin sodium Cezol (Deva Holding A.S., Istanbul, Turkey) was administered intravenously. For skin preparation, 10% povidone-iodine solution was used before the incision. Sternum surgical 3M™Ioban™ 2 antibacterial incise drape (3M Health Care, St. Paul, United States of America) was used. Median sternotomy was performed under general anesthesia to all the patients. Skin incision was made with a surgical scalpel. Subcutaneous tissues were cut with monopolar electrocautery (ErbeVIO® 300S; Erbe Elektromedizin GmbH, Tuebingen, Germany) in swift coagulation mode at a maximum energy setting of 60 watts. Before placing the sternum retractor, sterile surgical towels were placed on both sides of the sternum for protection. Bone wax was not used in the patients. CPB was performed by using aortocaval cannulation technique in all the patients following systemic heparin administration (300 IU/kg). Cardiac arrest was achieved by using hypothermic, hyperkalemic blood cardioplegia and topical hypothermia. Surgery was performed under moderate systemic hypothermia (32°C). CPB flow was maintained at 2.2–2.5 L/min/m^2^, mean perfusion pressure was maintained between 50 and 80 mmHg, and hematocrit level was maintained at 20–25% during CPB. Cardiac arrest was achieved by using intermittent antegrade infusion of cold blood cardioplegia. For the patients that had low EF, multivessel disease, or poor ventricular function, continuous retrograde cold blood cardioplegia infusion was used in addition to infusion of intermittent antegrade cold blood cardioplegia. Warm blood cardioplegia was infused in all the patients just before removing the cross-clamp. Left internal mammary artery was used for revascularization of the left anterior descending artery in all the patients who underwent coronary artery bypass grafting. Bilateral internal mammary artery was not used in any patient. All proximal anastomoses were performed by using side clamps.

### Sternal Closure Technique

**Fig. 1:**
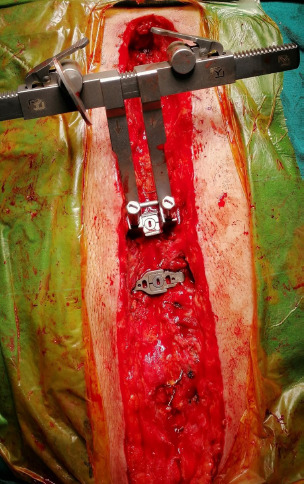
Sternal titanium plate application.

Monoflament stainless steel wire no.5 (Monowire; Boz, Ankara, Turkey) was used for sternal closure in all the patients. Two single transsternal wires were applied to the sternum manubrium. Two figure-of-8 parasternal steel wires were applied proximal, and two single transsternal and parasternal steel wires were applied distal to the corpus sterni. The wire sutures were tied tightly in the midline, and their ends were placed in the sternal tissue. Intercostal holes adjacent to the corpus sterni were opened with the help of electrocautery in the patients to whom sternal plating would be applied. Plates were placed next to the parasternal line in the patients that underwent closure with a titanium plate (Fixter; Yayla-Med, Ankara, Turkey). Distances were measured to correctly decide on the plate size. Titanium plates were placed in the closure apparatus and aligned parallel to the sternum, and the ends of the plates were placed in the intercostal holes. The closure apparatuses were brought closer to each other, the titanium plates were clamped together, and the sternal fixation was completed by turning the screw to the closing position (Figure 1). Tissue reapproximation was performed with three-layer continuous running closure in both sternal closure methods.

Closure technique and patient selection for sternal reinforcement was at the discretion of the surgeon on a case-by-case basis depending on the presence of risk factors. Titanium plate reinforcement was applied in the presence of two or more of the following causes: BMI ≥ 30kg/m^2^, advanced COPD, uncontrolled DM, and osteoporotic appearance of the sternum.

### Ventilator Management and Postoperative Antibiotic Therapy

In the postoperative period, remifentanil Ultiva® (GlaxoSmithKline Manufacturing S.p.A, Parma, Italy) was administered to each patient as an intravenous infusion. In the ICU, ventilator settings were set as synchronized intermittent mechanical ventilation mode, with tidal volume of 6-10 ml/kg, positive end-expiratory pressure of 5 cm/H_2_O, and respiratory rate of 12-16/min. By making necessary changes based on the blood gas monitoring results, remifentanil infusion was discontinued in the patients that had stable hemodynamics (without excessive bleeding < 50 ml/h) in the first postoperative hour. The patients had to be fully awake and able to move their upper and lower limbs easily and without signs of neurological deficit. Arterial blood gas values as pH > 7.35, PaCO_2_ < 40 mmHg, and PaO_2_ > 70 mmHg were required. Prior to extubation, chest X-ray was examined carefully to not to neglect pneumothorax, atelectasis, or severe pleural effusion. The physicianin charge of the cardiac surgery in the ICU was responsible for making the final decision to extubate the patient after a successful attempt of spontaneous ventilation and confirmation of normal neurological findings.

In all patients, 1 g intravenous cefazolin sodium treatment with 12-hour intervals was continued for another 24 hours after removal of mediastinal and/or thorax drain. Since fever developed after the third postoperative day, Tazocin® and Targocid® were given to three patients that were evaluated by the infectious disease’s clinic due to the diagnosis of bacterial pneumonia, ertapenem was given to one patient due to the diagnosis of urinary tract infection, and Tazocin® and teicoplanin were given to two patients due to the diagnosis of saphenous wound infection. No sternal complications were observed in any of these patients.

### Power Analysis

The power analysis of the study was performed with the G*Power 9.1.2 (Universitaet Kiel, Germany) program. A pilot study was conducted for the power analysis, and BMI values of the patients that were applied steel wire and titanium plate were used for calculation of the effect size. The effect size determined with the values of both groups was calculated as d=0.894. *T*-test was chosen as the test family, and the independent samples *t*-test was selected for the statistical analysis according to one-way table value. The minimum sample size for the groups was determined with a ratio of 2:1 as 35 and 70 — a total of 105 patients — by taking the margin of error as 5% and the power value as 0.95.

### Statistical Analysis

Statistical analyses of the study were performed with IBM Corp. Released 2017, IBM SPSS Statistics for Windows, version 25.0, Armonk, NY: IBM Corp. program. Descriptive statistics were presented as median (Q1-Q3) and frequency (percentage). Normality of the continuous variables was examined with Kolmogorov-Smirnov test. It was observed that the continuous variables were not distributed normally. Comparisons of the measured values of the groups created according to the operation types applied were performed with Mann-Whitney U test. Chisquare analysis was used to determine the relationships between the categorical variables. A logistic regression model was created in order to determine the factors affecting the operation types. Throughout the study, a *P*<0.05 value was considered statistically significant by taking the type-I error rate as 5%.

## RESULTS

**Fig. 2:**
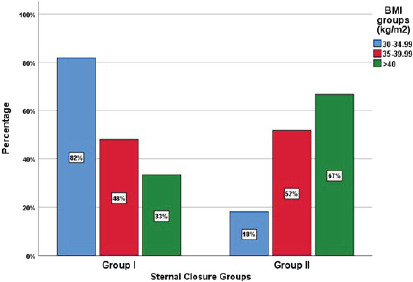
Sternal closure groups. BMI=body mass index.

**Fig. 3:**
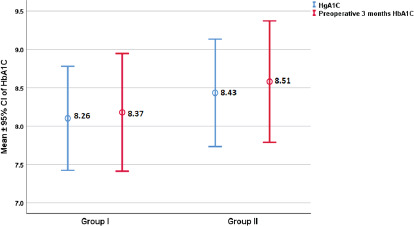
Hemoglobin A1C (HbA1C) levels of groups. CI=confidence interval; HbA1C=hemoglobin A1C.

The data of 316 patients that underwent an open-heart surgery between May 2018 and October 2021 were analyzed retrospectively. A total of 124 patients that had a BMI value ≥ 30 kg/m^2^ and underwent open-heart surgery were included in the study. There were 88 patients in group I (70.9%) and 36 patients in group II (29.1%). In our study, the percentage of male patients was 57.3%, and the percentages of male patients in group I and female patients in group II were found to be significantly higher (*P*<0.001). While the percentage of patients with BMI value ≥ 30-34.99 kg/m^2^ was 71%, the percentage of patients with BMI ≥ 35.00-39.99 kg/m^2^ was 21.8%. The BMI value of the remaining patient group was ≥ 40 kg/m^2^. The highest BMI value was measured as 48.24 kg/m^2^. While low BMI values were detected in group I, high BMI values were observed in group II (*P*<0.001) (Figure 2). The median age of the patients was calculated as 63 (33-85) years. Operation types and emergency surgical intervention rates did not difer significantly between the groups.Titanium plate usage was found to be significantly higher in patients with DM (*P*<0.001). However, no significant difference was found between hemoglobin A1C levels of the patients with DM between the groups (*P*=0.956). Again, no statistical significance was found in the hemoglobin A1C levels of the patients with DM, which were measured three months before and just before the operation (Figure 3). Also, COPD and smoking did not difer significantly between the groups (*P*=0.082, *P*=0.507). Postoperative mortality rate was found slightly higher in group II, but it was not statistically significant (*P*=0.409). The rates of non-infectious sternal wound complication and deep sternal wound infection were found slightly higher in group II, and the rate of sternal dehiscence was found higher in group I, but there was no statistically significant difference between the groups (Table 1). European System for Cardiac Operative Risk Evaluation (EuroSCORE) values were found to be significantly higher in group II (*P*=0.003). EF, CPB, ventilation time, duration of ICU and hospital stays, and use of blood products did not difer significantly between the groups. Yet, preoperative hematocrit levels were found to be significantly lower in group II (*P*=0.028) (Table 2).

**Table 1:** Demographic and clinical characteristics of the patients.

	Group I	Group II	Total	
	n (%)	n (%)	n (%)	*P*-value
Sex				
Male	61 (69.3)	10 (27.8)	71 (57.3)	< 0.001*
Female	27 (30.7)	26 (72.2)	53 (42.7)	
BMI (kg/m^2^)				
≥30-34.99	72 (81.8)	16 (44.4)	88 (71.0)	< 0.001*
≥35-39.99	13 (14.8)	14 (38.9)	27 (21.8)	
≥40	3 (3.4)	6 (16.7)	9 (7.3)	
Type of operation				
AVR	5 (5.7)	1 (2.8)	6 (4.8)	0.286
CABG	74 (84.1)	30 (83.3)	104 (83.9)	
Of-pump CABG	6 (6.8)	2 (5.6)	8 (6.5)	
MVR	3 (3.4)	3 (8.3)	6 (4.8)	
Emergency operation				
None	83 (94.3)	34 (94.4)	117 (94.4)	0.978
Yes	5 (5.7)	2 (5.6)	7 (5.6)	
DM				
None	44 (50.0)	3 (8.3)	47 (37.9)	< 0.001*
Yes	44 (50.0)	33 (91.7)	77 (62.1)	
COPD				
None	61 (69.3)	19 (52.8)	80 (64.5)	0.082
Yes	27 (30.7)	17 (47.2)	44 (35.5)	
Smoker				
None	77 (87.5)	33 (91.7)	110 (88.7)	0.507
Yes	11 (12.5)	3 (8.3)	14 (11.3)	
Postoperative mortality				
None	84 (95.5)	33 (91.7)	117 (94.4)	0.409
Yes	4 (4.5)	3 (8.3)	7 (5.6)	
Sternal complication				
None	80 (90.9)	32 (88.9)	112 (90.3)	0.517
Non-infectious sternal wound complication	4 (4.5)	2 (5.6)	6 (4.8)	
Sternal dehiscence	3 (3.4)	0	3 (2.4)	
Deep sternal infection	1 (1.1)	2 (5.6)	3 (2.4)	

* Significant at 0.05 level according to Chi-square test

AVR=aortic valve replacement; BMI=body mass index; CABG=coronary artery bypass grafting; COPD=chronic obstructive pulmonary disease; DM=diabetes mellitus; MVR=mitral valve replacement

**Table 2:** Perioperative and postoperative characteristics of patients.

	Group I	Group II	Total	
	Median; Q1-Q3			*P*-value
Age, years	63; 55-69	64; 59-70	63; 56-69	0,343
BMI, kg/m^2^	32;30.5-34.3	35; 32.8-38.7	33; 30.8-35.7	< 0.001*
EuroSCORE	1.35; 0.83-2.10	2.03; 1.33-2.63	1.46; 1.00-2.35	0.003*
EF	57; 50-65	55; 45-63.7	55; 48.5-65	0.511
Hematocrit, %	39.3; 35.8-43.7	37.1; 33.7-41.3	38.5; 35.2-43.4	0.028*
CPB time, min	119; 98-142	117; 93-146	119; 56-142	0.920
ES, n	1; 1-2	2; 1-2.75	1; 1-2	0.065
FFP, n	1; 1-2	1; 1-2	1; 1-2	0.806
Ventilation time, h	4; 4-6	5; 4-7.25	5; 4-6	0.139
ICU time, days	3; 2-4	3; 2-3.75	3; 2-4	0.912
Hospitalization time, days	8; 7-10	8; 7-11	8; 7-10	0.858

* Significant at 0.05 level according to Mann-Whitney U test

BMI=body mass index; CPB=cardiopulmonary bypass; EF=ejection fraction; ES=erythrocyte suspension; EuroSCORE=European System for Cardiac Operative Risk Evaluation; FFP=fresh frozen plasma; ICU=intensive care unit

A univariate logistic regression model was created in order to determine the factors that affect the decision of using titanium plate by accepting group I — the group who underwent steel wire closure — as a reference. Significant factors were obtained in the third stage of the model, which was established by using the forward stepwise logistic regression, and the model was found to be significant (Hosmer-Lemeshow X^2^=4.726; *P*=0.786). Goodness-of-fit values of the model were found to be high (-2LL=107.28; R^2^ Nagelkerke=0.411; Omnibus X^2^=42.125 and *P*<0.001). It was observed that increasing BMI values (odds ratio [OR]: 1.18), female gender (OR: 4.366), and presence of DM (OR: 7.352) contributed significantly to the model (Table 3).

**Table 3:** Factors affecting titanium plate.

	Beta	*P*-value	OR	95% CI
BMI	0.165	0.027*	1.180	1.019-1.366
Sex (female)	1.476	0.003*	4.366	1.680-11.363
DM	1.992	0.003*	7.352	1.937-27.777
EuroSCORE	0.077	0.782	1.080	0.236-5.216
Hematocrit	0.014	0.907	1.010	0.147-6.108

* Significant at the 0.05 level according to the two-category logistic regression analysis

BMI=body mass index; CI=confidence interval; DM=diabetes mellitus; EuroSCORE=European System for Cardiac Operative Risk Evaluation;OR=odds ratio

Sternal complication rates in patients with BMI ≥ 35 kg/m^2^ were evaluated. The distribution of complications was found to be different in the BMI ≥ 35.00-39.99 kg/m^2^ and BMI ≥ 40 kg/m^2^ groups (*P*=0.003). The rate of development of complications was found to be higher in the patient group with BMI ≥ 40 kg/m^2^. In group I, deep sternal wound infection was observed only in the patient group with BMI ≥ 40 kg/m^2^ (n:1, *P*=0.028). In group II, no other complication was observed except for deep sternal wound infection. Both patients that had deep sternal wound infection in group II had a BMI value ≥ 40 kg/m^2^ (Table 4). Of the three deep sternal wound infection complications detected in both groups, one was positive for methicillin-resistant *Staphylococcus aureus*; yet, two did not grow any organisms in culture. The methicillin-resistant *S. aureus* was treated intravenously with meropenem and teicoplanin for four weeks, and the remaining two cases were de-escalated to treatment with Tazocin® and Targocid®. No significant relationship was found between the sternal closure techniques and sternal complications (*P*=0.404) (Table 5).

**Table 4:** Sternal complication rates in patients with body mass index (BMI) ≥ 35 kg/m^2^.

BMI	≥ 35-39.99 kg/m^2^	≥ 40 kg/m^2^	Total	
	n (%)	n (%)	n (%)	*P*-value
**Sternal complication**				
None	24 (89.9)+	5 (55.6)+	29 (80.6)	0.003*
Non-infectious sternal wound complication	2 (7.4)	0	2 (5.6)	
Sternal dehiscence	1 (3.7)	1 (11.1)	2 (5.6)	
Deep sternal infection	0++	3 (33.3)++		
**Group I**	**n (%)**	**n (%)**	**n (%)**	***P*-value**
**Sternal complication**				
None	10 (76.9)	1 (33.3)	11 (68.8)	0.028*
Non-infectious sternal wound complication	2 (15.4)	0	2 (12.5)	
Sternal dehiscence	1 (7.7)	1 (33.3)	2 (12.5)	
Deep sternal infection	0+	1 (33.3)++	1 (6.3)	
**Group II**	**n (%)**	**n (%)**	**n (%)**	***P*-value**
**Sternal complication**				
None	14 (100)+	4 (66.7)+	18 (90)	0.026*
Deep sternal infection	0++	2 (33.3)++	2 (10)	

* Significant at *P*<0.05 level according to chi-square analysis

+,++The difference between the column ratios of similar symbols is significant at the 0.05 level

**Table 5:** Sternal complication rates of patients with body mass index ≥ 35 kg/m^2^ by operation groups.

	Group I	Group II	Total	
Sternal complication	n (%)	n (%)	n (%)	*P*-value
None	11 (68.8)	18 (90.0)	29 (80.6)	0.404*
Non-infectious sternal wound complication	2 (12.5)	0	2 (5.6)	
Sternal dehiscence	2 (12.5)	0	2 (5.6)	
Deep sternal infection	1 (6.3)	2 (10)	3 (8.3)	

* Significant at the *P*<0.05 level according to the chi-square analysis

## DISCUSSION

Sternal complications due to median sternotomy can be seen at a rate of 0.5-2.5% after all open-heart surgeries^[1,3,12]^. Obesity is also a well-known important risk factor for the development of sternal complications^[3,5,6]^. As the incidence of obesity in the community increases, the number and percentage of morbidly obese patients undergoing open-heart surgery is also increasing^[2,13,14]^. In our study, the obesity rate in patients that underwent open-heart surgery was 39%, and the percentage of patients with BMI ≥ 35 kg/m^2^ was found to be 11.3%.

Although many techniques and interventions have been suggested for the surgical treatment of sternal complications, no consensus has been reached on the superiority of any of these techniques, and no closure technique that can prevent the development of sternal complications in the presence of risk factors has been defined yet.

In a retrospective study conducted by Liao et al.^[15]^ on patients with BMI ≥ 35 kg/m^2^, the results of patients that underwent single primary xiphoid transverse titanium plate reinforcement for primary sternal closure were compared with the results of patients that underwent cardiac surgery without sternal plate reinforcement. They found no difference in sternal dehiscence, wound drainage, mediastinitis, and 30-day mortality rates. Tulugan et al.^[3]^ also compared patients whose manubriosternal joint was applied plate reinforcement with patients whose manubriosternal joint was not applied plate reinforcementin a study they conducted on patients with BMI ≥ 35 kg/m^2^. They found no difference between sternal dehiscence, deep sternal wound infection, and non-infectious sternal complication. In both studies, no significant difference was found between the operation types, 30-day mortality, and length of ICU and hospital stay. In a multicenter, randomized, and blinded study conducted by Allen et al.^[16]^, sternal complication rates in sixth months were compared between patients that were performed wire cerclage and patients that were performed rigid plate fixation. Sternal wound complications were found to be less in the sternal plate applied group. In our study, no statistical difference was found between the groups in terms of operation types (*P*=0.286) and the duration of ICU (*P*=0.912) and hospital stay (*P*=0.858). Mortality rates were slightly higher in group II, but no statistically significant difference was found (*P*=0.409). This height was attributed to the higher BMI rates, higher DM rate and EuroSCORE levels, and lower preoperative hematocrit values in group II patients in whom steel wire combined with titanium plates was used.

Presence of risk factors for the development of sternal dehiscence may guide clinicians to sternal reinforcement in primary closures. Dell’Amoreet al.^[17]^ identified obesity, DM, COPD, and surgical re-exploration as risk factors for the development of sternal complications. Similar risk factors were also reported by Noohetal.^[18]^. Enhanced sternal stabilization (Class of Recommendation: IIb, Level of Evidence: C) is recommended for mediastinitis and sternal dehiscence in high-risk patients by The Society of Thoracic Surgeons Clinical Practice Guidelines. In addition, glycemic control (Class of Recommendation: IIa, Level of Evidence: B) and smoking cessation (Class of Recommendation: I, Level of Evidence: C) were recommended in the same study^[11]^. In our findings, it was found that increased BMI rate (OR: 1.180, 95% confidence interval [CI]: 1.019-1.366), female gender (OR: 4.366, 95% CI: 1.680-11.363), and the presence of DM (OR: 7.352, 95% CI: 1.937-27.777) increased the rate of application of sternal reinforcement in patients.

Snyder et al.^[19]^ compared the patients that had a BMI > 30 kg/m^2^ and high risk of sternal complications and received plate closure with those that had a BMI > 30 kg/m^2^ and high risk of sternal complications and received wire closure. Early wound complications were found to be significantly lower in the plate group (*P*=0.067, Fisher’s exact test, *P*=0.035). However, there was no difference determined between the rates of late sternal wound complications. Recent meta-analyses have shown that rigid plate fixation may reduce sternal complications in high-risk patients^[20,21]^. In our study, we did not detect any non-infectious sternal wound complications or dehiscence, especially in the patients that had BMI ≥ 35 kg/m^2^ and were applied sternal reinforcement by us. Deep sternal wound infection rates did not show statistical significance between the two groups.

### Limitations

A major limitation of this study is selection bias, in which patients at higher risk of sternal complications are more likely to receive sternal plating, resulting in a type II error. Considering the limitations of a retrospective, non-randomized study, the complication rates might have been higher if the plate had not been used in the patients that had their sternum closed with titanium plates, and thus it would mask a potential difference. However, the variance in how the decision of sternal closure for each patient was made by different surgeons essentially randomized the population. More multicenter randomized controlled trials are needed to determine the patient groups to be applied primary sternal plating.

## CONCLUSION

In conclusion, there was no difference observed in sternal complication rates in the patients that had a BMI ≥ 30 kg/m^2^ and underwent titanium plate-supported sternal closure. However, the lower incidence of complications along with the absence of non-infectious sternal wound complications and sternal wound dehiscence in the group of patients that had a BMI ≥ 35 kg/m^2^ and underwent plate-supported sternal closure strengthened the idea that plate-supported sternal closure can prevent sternal complications in high-risk patients. As a result of this study and approximately three and a half years of experience, primary sternal closure with plate reinforcement has been adopted and started to be routinely performed on patients that have a BMI value ≥ 35 kg/m^2^ and risk factors for sternal dehiscence, such as DM, COPD, osteoporosis, and chronic kidney failure, in our clinic.
